# Microstructural Inhomogeneity of Electrical Conductivity in Subcutaneous Fat Tissue

**DOI:** 10.1371/journal.pone.0117072

**Published:** 2015-03-03

**Authors:** Ilja L. Kruglikov

**Affiliations:** Wellcomet GmbH, Karlsruhe, Greschbachstrasse 2–4, 76229 Karlsruhe, Germany; University of Nebraska Medical Center, UNITED STATES

## Abstract

Microscopic peculiarities stemming from a temperature increase in subcutaneous adipose tissue (sWAT) after applying a radio-frequency (RF) current, must be strongly dependent on the type of sWAT. This effect is connected with different electrical conductivities of pathways inside (triglycerides in adipocytes) and outside (extra-cellular matrix) the cells and to the different weighting of these pathways in hypertrophic and hyperplastic types of sWAT. The application of the RF current to hypertrophic sWAT, which normally has a strongly developed extracellular matrix with high concentrations of hyaluronan and collagen in a peri-cellular space of adipocytes, can produce, micro-structurally, a highly inhomogeneous temperature distribution, characterized by strong temperature gradients between the peri-cellular sheath of the extra-cellular matrix around the hypertrophic adipocytes and their volumes. In addition to normal temperature effects, which are generally considered in body contouring, these temperature gradients can produce thermo-mechanical stresses on the cells’ surfaces. Whereas these stresses are relatively small under normal conditions and cannot cause any direct fracturing or damage of the cell structure, these stresses can, under some supportive conditions, be theoretically increased by several orders of magnitude, causing the thermo-mechanical cell damage. This effect cannot be realized in sWAT of normal or hyperplastic types where the peri-cellular structures are under-developed. It is concluded that the results of RF application in body contouring procedures must be strongly dependent on the morphological structure of sWAT.

## Introduction

According to the widely accepted perception of subcutaneous white adipose tissue (sWAT), the dynamic modulation of its structure and volume is mainly connected with the processes of lipogenesis and lipolysis, together with slow dynamic processes of adipose tissue renewal, having the characteristic time of approximately 10 years. Consequently, sWAT appears in aesthetic applications as spatially homogeneous and inert structure, demonstrating slow reactions to different metabolic or environmental modulations.

Quite contrary to this notion, a vast amount of new experimental and theoretical results attained during recent years, clearly demonstrated that architectural peculiarities and micromechanical properties of sWAT are significantly determined, not by adipocytes, but more by the extracellular matrix (ECM) of this tissue. It was shown that ECM in sWAT can contain an increased amount of hyaluronan in peri-cellular areas of adipocytes, which can lead to high local water retention in these regions [1,2]. ECM of sWAT can also demonstrate the local inter- and peri-cellular fibrose structures which contain different types of collagen [3]. These structural properties of ECM are strongly dependent on the morphological type of sWAT differing in hyperplastic and hypertrophic sub-types of this tissue. The description of sWAT as a homogeneous tissue, which is generally, indirectly assumed in aesthetic medicine, can thus lead to serious mistakes in proposed biophysical mechanisms of different non-invasive aesthetic procedures. Consequently, in terms of the theory of these applications, the point of interest must be shifted from the average values of absorbed energy towards the direction of the microscopically inhomogeneous distribution of this energy in sWAT.

Recently, it was demonstrated that the mechanical properties of sWAT are mainly connected to its peri-cellular fibrotic structure, produced around the adipocytes to restrict their expansion [4–7] and are much less dependent on the properties and the structure of inter-cellular fibrosis in this tissue. This mechanical microstructure of ECM seems to be so important for sWAT, that it was even recently supposed that it can be mainly responsible for the differentiation between healthy and unhealthy obesity [6]. The mechanical properties of sWAT, such as Young’s modulus [4] and tensile strength [6] are actually believed to be mainly dependent on the peri-cellular structure containing collagens of the types IV and VI. For example, the sWAT in knockout mice which does not produce collagen VI demonstrates the tensile strength which is approximately only 50% of this value in normal sWAT [6], making such structurally modulated tissue much less rigid. Whereas the modulation of the cell's size distribution of adipocytes has almost no influence on the mechanical characteristics of sWAT, which is mainly determined by the average size of adipocytes in a given sWAT region, these properties can be strongly affected by appearance of dead cells and rigid inclusions e.g. in a form of clusters of small adipocytes [7]. Based on this model, it was predicted in [7] that under other equal conditions, the application of different physical factors that mainly cause adipocytes’ death or activation of lipolysis, must demonstrate qualitatively different modulations of mechanical properties of sWAT—the result which is of primary importance in obesity research as well as in all aesthetic body contouring procedures.

According to this formalized sWAT description, one can therefore conclude that not only the pure mechanical but also the electrical, thermic and the thermo-mechanical characteristics of sWAT must consequently be dependent on its ECM structure. In aesthetic applications, it is generally assumed, that the heating of sWAT with the application of different physical modalities (such as radio-frequency current, ultrasound, light, etc.) must lead to quasi-homogeneous distribution of absorbed energy in the tissue to receive the best clinical results. Recently however, it was proposed that heating of sWAT with a radio-frequency (RF) current can lead to a non-homogeneous temperature distribution in a tissue's volume, connected with selective energy absorption in its septae filament structures [8]. The intercellular fibrotic structure of septae consists mainly of fibrillar collagens of the types I and III and is coarse-mesh with a typical unit size (distance between septae) of several millimeters. However, the peri-cellular structure around the adipocytes containing the non-fibrillar collagens of the types IV and VI with characteristic sizes of a single unit of 50–150 μm corresponding to the typical sizes of adipocytes, can be more important for the heating procedure. These structures can selectively conduct and absorb the RF current causing overheating at the surfaces of adipocytes, thus leading to a microscopically inhomogeneous temperature distribution in sWAT which was ignored in all former theoretical interpretations of RF applications.

In this article we will apply the micro-mechanical model of sWAT discussed in [7] to describe a non-invasive application of RF current to this tissue.

## Thermo-Mechanical Effects in sWAT

Temperature variation can cause different metabolic and structural changes in sWAT, which can affect its mechanical properties and modify its volume in the long- or short-term. We will not discuss the processes connected with metabolic reactions (such as stimulation of lipolysis) further in this article; these processes are normally transient and cannot demonstrate any significant clinical results in a short timeframe [9].

### Collagen Shrinkage Phenomenon and its Possible Role in sWAT Modulation

Important structural modifications to the tissue during its heating can be connected with conformational changes of proteins in membranes of adipocytes as well as in its ECM structure. The first modification can lead to the transient increase of permeability of cell membranes, or, in the case of denaturation of these proteins, to a cell death. Increased permeability of adipocytes was, for example, described in [10] after the mild homogeneous temperature increase in sWAT during the application of non-focused external ultrasound. Modification of the ECM structure is believed to be the main target of treatments which belong to the group of “skin tightening” methods in aesthetic treatments, where it is generally assumed that denaturation of collagen (also known as “collagen shrinkage”) under heating does not cause any cell death but leads to the increase of partial volume occupied by collagen molecules and thus to some “tightening”-effect in the tissue.

Collagen “shrinkage” was well investigated both *in vitro* and *in vivo*. It is known that this effect does not only depend on the temperature applied, but also on the exposure time, quality and orientation of collagen fibers, age and pre-history of collagen, water content in the tissue, etc. It was originally assumed that collagen shrinkage could be mainly responsible for treatment results observed in such specific applications of RF current as capsulorrhaphy and thermokeratoplasty [11]. It is however very doubtful that this effect plays any important role in different non-invasive (and even in some minimal invasive) methods applied for aesthetic skin modification or in body contouring. The reasons for this were discussed in detail [12] and can be shortly summarized as follows:

1. The temperatures and/or exposure times needed for collagen denaturation are far over the levels normally applied in conventional non-invasive body contouring procedures;
2. The appearance of collagen shrinkage *in vivo* must be accompanied by massive cell death which normally already takes place at temperatures over 43°C and which is generally not observed after non-invasive RF applications;
3. The appearance of denatured collagens in the tissue must immediately activate the process of their enzymatic degradation, inducing the production of specific matrix metalloproteinases (especially of gelatinases) and thus dramatically reducing the potential timeframe for positive clinical results;
4. Massive local collagen shrinkage in the tissue must lead to a significant decline in tissue stiffness; this effect would be generally opposite to the expected effect of tissue tightening.

Therefore, collagen shrinkage cannot be the main effect responsible for the tissue modification after local heating of sWAT with RF currents. Since such (if any) increase in stiffness can sometimes be observed in the short term after RF heating, the most reasonable explanation for this would be the local transient endogenous hyaluronan production in the heated area connected with a local water retention in sWAT, increasing the tissue turgor [9]. This is normally a transient effect with a relatively short recovery time.

### Role of Microstructural Heterogeneity of Electrical Conductivity in sWAT

Heating procedures based on the application of different types of energy can lead to various temperature distributions in sWAT, whether for producing more or less homogeneously or strongly selective energy absorption. The latter could be the case, for example, by application of RF current and would be theoretically connected with a high difference in electrical conductivities between ECM structures and triglycerides in sWAT.

The main mass of sWAT consists of triglycerides which have very low electrical conductivities and thus can be mainly characterized as dielectrics. Triglycerides are gathered in spatially limited compartments (adipocytes) surrounded by cell membranes. These membranes are reinforced by peri-cellular collagen structures containing collagens IV and VI [4,6,7]. If the electrical conductivity of the peri-cellular structure around an adipocyte is much higher than the electrical conductivity of triglycerides inside of it, the RF current must flow almost exclusively through the ECM and not through the cells’ volumes. Such a selective increase in the local current density in relatively thin layers around the cells can be named current “channeling”. It can be assumed, that the effect of “channeling” will lead to selective heating of adipocyte’s surfaces with production of much higher absolute temperatures as well as significantly higher temperature gradients near these surfaces. It is necessary to discuss this item in further detail, since this perception can completely change the interpretation of the RF effect on the sWAT, as well as significantly modify the priorities in the field of non-invasive body contouring with the application of RF.

Based on the abovementioned, sWAT can be electrically described as a large number of small insulating spheres (adipocytes) one part of which (1-*p*) has insulating surfaces and another part (*p*) covered with conducting material (peri-cellular structure). It is commonly known that such a system demonstrates a percolating behavior, i.e. by low values of *p* < *p*_*cr*_ its conductivity is zero, by *p* > *p*_*cr*_ conductivity increases with *p* and it reaches its maximum value at *p* = 1 [13]. The value of *p*_*cr*_ in different percolation systems was assessed to be approximately 0.30. Consequently, if only a small part of cells in sWAT are covered with peri-cellular structures, its effective conductivity can be very low. Indeed, in this case the majority of conducting structures will build the closed spatially limited clusters, which do not allow the electrical current to flow through the tissue. In the opposite case, where almost all adipocytes are covered with conductive peri-cellular structures, sWAT can be described as closed-cell foam where almost all unit cells contact electrically with their neighbors. Since the peri-cellular structure around the adipocytes is the hallmark of hypertrophic fat tissue, one can immediately conclude that the effect of RF current in sWAT must be strongly dependent on the type of sWAT (being more pronounced in sWAT of hypertrophic than in sWAT of hyperplastic type). This could be an important reason for the commonly known significant inter-regional and inter-patient variations in results of RF application.

It is obvious that adipocytes in sWAT do not have the same size and can be characterized by some cell size distribution [7]. From the percolation model described above, one can predict that the large cells (with better peri-cellular structures) would be preponderant for the electrical conductivity of sWAT in the case of *p* ~ 1; this result is well known from experiments with a mixture of insulated and coated spheres [14].

In the following, we will consider the idealized model of sWAT as a closed-cell foam [4,7] containing only the adipocytes surrounded by peri-cellular structures, i.e. the case of *p*≅1. Effective electrical conductivity of such closed-cell foam ($\tilde{\lambda }$) is dependent on the electrical conductivity of the peri-cellular structure enveloping the cell walls (*λ*_*s*_), on the electrical properties of material filling the single cells (*λ*_*m*_), on the form and the size of a unit cell (single adipocyte) and on the relative density of the foam ($\tilde{\rho }/\rho_{s}$), where $\tilde{\rho }$ is the effective density of the foam and *ρ*_*s*_ is the material density of the peri-cellular structure. For the closed-cell foam of low relative density containing isotropic cells which are filled with absolute insulated material (*λ*_*m*_ = 0) the effective electrical conductivity of sWAT can be approximated by the following equation:

$$\tilde{\lambda }/\lambda_{s}≅{\left(\frac{\tilde{\rho }}{\rho_{s}}\right)}^{\gamma }$$ (1)

where *γ* normally has the values between 1.5 to 2.0 [15]. The value of $\tilde{\lambda }$ was measured for different current frequencies to be in the interval of 0.02–0.05 S/m [16]. Dependent on the adipocytes’ size, the value of $\tilde{\rho }/\rho_{s}$ can be assessed to be up to 0.20, with typical values of 0.10–0.15 [4].

For further estimations, we will set $\tilde{\lambda }$ = 0.02 S/m (which corresponds to the RF frequency of 1 MHz), $\tilde{\rho }/\rho_{s}$ = 0.15, and γ = 1.5. From Equation (1), if the RF current predominantly flows through the peri-cellular fibrotic structures of ECM in sWAT, the electrical conductivity of this structure can be assessed to have a value of approximately *λ*_*s*_ = 0.34 S/m. For the same value of $\tilde{\lambda }$, the sWAT with relative foam densities of 0.10 and 0.20 will have the electrical conductivity of its peri-cellular structure of 0.63 S/m and 0.22 S/m, respectively. As it will be demonstrated below, these variations can significantly modify the temperature profile in sWAT during RF heating. The value of *λ*_*s*_ cannot be modelled or measured independently since the peri-cellular structure is complex and not exactly known, but the values obtained in these estimations are in good agreement with electrical conductivity of the viable skin which is in the order of 0.4 S/m [16] and which also consists of the mixture of collagens, glycosaminoglycans and water. This indirectly supports the electrical model of sWAT described above.

It is difficult to estimate the electrical conductivities of triglycerides and of peri-cellular structures in different types of sWAT, since these measurements are very sensitive to the structural variations of the tissue. For example, the values of electrical conductivities can be changed by orders of magnitude with increased water content. Since the content of intra-cellular water in adipocytes is very low, we can neglect it, further estimating the pure triglycerides. In addition, to make the rough estimations, we will avoid the discussion about the influence of the mixture of different triglycerides on the value of electrical conductivity, and instead use the electrical conductivity of pure glycerol which is approximately of 6.4x10^-8^ S/cm at 25°C. Changes in the water content in the mixture glycerol-water can modify this value by an order of magnitude. However, in any case this value is much less than the value of *λ*_*s*_ estimated above.

The Electrical conductivity of pure collagens was measured to be 2.5x10^-8^ S/cm by low and of 2.0x10^-5^ S/cm by high water content. These values are also much smaller than the value of *λ*_*s*_ assessed above, which means that collagen alone does not define the effective electrical conductivity of the peri-cellular structure.

### Homogeneous vs. Inhomogeneous Temperature Increase in sWAT

Two different scenarios are theoretically possible:

*1. “Dry” ECM environment*

In this case, the electrical conductivities would be similar inside and outside of adipocytes and there is no prevalence in the pathways for RF current in sWAT. Here, the temperature distribution in sWAT must be quasi-homogeneous. This would be the case for sWAT with low content of peri-cellular structures, e.g. for a hyperplastic type of sWAT. Effective conductivity of this system must however be low, which will significantly reduce the current's density and consequently the heating effect in such a system.

*2. “Moist” ECM environment*

In this case, the electrical conductivities would be very different for the pathways through and around the adipocytes, whereas the pathway through the ECM will prevail over the pathway through adipocytes. This will cause the concentration of the current flow near the cells’ surfaces, producing the effect of current “channeling”. This effect must cause microscopically inhomogeneous temperature distribution in sWAT producing the selective heating and high temperature gradients on the surfaces of adipocytes covered with peri-cellular structures.

The realization of these scenarios are significantly dependent on the type of sWAT and its pre-history. While hypertrophic fat tissue can accumulate hyaluronan near the adipocytes’ surfaces [1,2], its water content must be correspondingly higher as in sWAT of the normal or of the hyperplastic type [17]. This high local water retention in sWAT is not only responsible for the “washout” phenomenon in such tissue, demonstrating a correlation of sWAT volume reduction with water loss [18], but it can also contribute to the shift of RF current flow from intra- to extra-cellular pathways. Production of peri-cellular fibrotic structures around the hypertrophic adipocytes, which is actually considered to be the hallmark of hypertrophic sWAT [3], would additionally shift the pathways into the direction of extra-cellular current flow. Consequently, the RF application (by the same current densities near the electrodes) must demonstrate different clinical results in sWAT of hypertrophic and of normal/hyperplastic types.

Whereas a more or less homogeneous mild temperature increase in sWAT by a short-termed sub-lethal application of heating cannot cause any significant modification of sWAT volume excluding the local oedema production (the case of “dry” ECM), the situation can be very different by selective heating of adipocytes’ surfaces (the case of “moist” ECM). In this case, much higher microscopic local temperatures (“hot surfaces”) can be achieved by applying the same amount of RF current to sWAT. Because of its special geometry (surface heating of spherical cells), such a temperature increase can even cause the thermo-mechanical reactions of adipocytes which are very different from those in the case of quasi-homogeneous temperature distribution in sWAT. As mentioned, the largest adipocytes will be preponderant for the electrical conductivity in the mixture of adipocytes of different sizes, which must produce an additional source for the inhomogeneous microscopic temperature distribution in sWAT, supposing the large cells will be more heated at their surfaces than the small ones.

A physical description of this system is given in *Appendix*. As demonstrated there, an average temperature increase in electrically quasi-homogeneous sWAT (by application of RF current with typical current densities during a short time corresponding to the adiabatic heating, i.e. heating without heat and mass exchange) is low and can reach only 1–2 K. This temperature increase cannot really modify the cell’s or the tissue’s structure. At the same time, a temperature increase in a peri-cellular structure around an adipocyte (because of the current “channeling” effect) can be approximately 6 times higher and can thus reach the values over 10 K (see Equation (A.4) and the following discussion) which will be enough to cause the biological reaction.

If this temperature jump takes place during the first several milliseconds of the RF application, which is too short for the thermal diffusion to re-distribute the heat in the tissue and which corresponds the characteristic adiabatic time of sWAT (see *Appendix*), high temperature gradients can be produced near the cells’ surfaces. A longer application of RF current will cause a further increase in the absolute average temperature in the tissue until this process is compensated by a tissue perfusion effect in Equation (A.1). At the same time, the difference between the temperatures on the surfaces and inside of adipocytes will be limited by the processes of thermal diffusion. This means that inhomogeneity of the temperature distribution in sWAT with building of temperature gradients will only be produced during the first several milliseconds of RF application.

For the case of *p* < 1, which means that not all adipocytes have the conducting peri-cellular structures, additional spatial inhomogeneity of temperature distribution in sWAT will arise. If the cells coated with peri-cellular structures can build the clusters, these spatially limited structures will have higher temperatures than the neighboring clusters of adipocytes without such a coating. This effect can produce another scale of temperature inhomogeneity as was described above on the single cell level and this effect will be considered elsewhere.

Strong microscopic temperature gradients can cause thermo-mechanical stress near the cell’s surface. The maximum mechanical stress in a cell subjected to heating with a constant surface temperature is described by Equation (A.8). The maximum temperature jump at the cell surface which does not cause the mechanical fracturing of the cell can be estimated from Equation (A.9). In the case where the maximum mechanical stress in the cell, $\sigma_{r}^{max}$, will reach the critical value of the material tensile strength, σ_*f*_, the cell will rupture. This rupturing can take place at the cell surface (as in the model described in *Appendix*) or inside the cell (as it would be the case for the constant heat flux into the cell by its irradiation). Thus Equation (A.9) gives the possibility to estimate the probability of thermo-mechanical cell damaging. Whereas this mechanism seems to be unrealistic for the intact sWAT, demanding a temperature of over 6.000 K, it can be theoretically realized for sWAT treated with high deformation rates, which can significantly increase its Young’s modulus. Such a modification of mechanical modules of sWAT was earlier described in [19].

## Discussion

The point of interest in sWAT is shifted more and more from adipocytes and triglycerides into the direction of its ECM. ECM structures and functions vary significantly in different types of sWAT and are actually believed to be substantially responsible for the mechanical characteristics of sWAT [4–7]. It was even recently assumed that variations in the micro-architecture of ECM, especially in the so-called peri-cellular structures coating the single adipocytes, can be an important hallmark to differentiate between the healthy and unhealthy obesities [6].

Peri-cellular structures only occupy a fractional part in sWAT which can be estimated to be less than 0.1–0.2 of the whole volume. Because of this, one would intuitively neglect the influence of these structures on different physical properties of the whole sWAT. However, this can lead to incorrect conclusions. For example, the mechanical modules of peri-cellular structures are much higher than the corresponding values for triglycerides in adipocytes [7]; consequently, they can dominate the integral physical properties of sWAT even by their small partial weighting.

At the same time, not only the mechanical but also the electrical and the thermic properties of ECM and those of adipocytes are very different. So, the electrical conductivity of peri-cellular structures must be significantly higher than the corresponding electrical conductivity of adipocytes. Consequently, the sWAT must be microscopically and not only mechanically, but also electrically, strongly inhomogeneous. Such microscopic inhomogeneity must cause the re-distribution of current densities in sWAT volume concentrating the current flow in thin peri-cellular layers near the cell surfaces. The last phenomenon can lead to selective heating of adipocytes’ surfaces, which seriously contradict the actual existing description of sWAT-heating by RF currents. As it is shown in *Appendix*, such microscopic temperature inhomogeneity will be produced during the first milliseconds of RF application, corresponding to the typical adiabatic times of sWAT heating.

To realize this possibility, the electrical conductivity of the peri-cellular sheath around adipocytes must be much higher than that of triglycerides inside the adipocytes. Only in this case will the temperature increase in sWAT be very heterogeneous, producing the high temperature gradients between the “hot” spots (adipocyte’s surfaces) and the “cold” areas (adipocytes’ volumes). As it was discussed above, this can be the case if sWAT has the strong peri-cellular fibrotic structure with high water content. These conditions are met only in sWAT of a hypertrophic type [7,17]. Reduction of either collagen or hyaluronan/water content in ECM of the normal or hyperplastic type of sWAT must significantly reduce the electrical conductivity of the peri-cellular adipocyte’s sheath, with the consequent re-distribution of current densities between ECM and the adipocyte’s volume. This re-distribution of the current will reduce the local superficial heating of adipocytes, decreasing the temperature gradients and making the temperature distribution in sWAT more homogeneous. The latter can however substantially modify the effect of RF application in hyperplastic sWAT compared to hypertrophic sWAT. Thus, the reaction of sWAT of different morphological types to the same amount of RF current can be very different, being more pronounced in sWAT of a hypertrophic type.

High temperature gradients developed on the surfaces of adipocytes have the characteristic spatial scales of several μm and can produce high local thermo-mechanical stresses, which can even cause mechanical damage to the cells. The required local temperature increase for the static (not deformed) sWAT is very high and can reach several thousand degrees of K. This mechanism can be realized at much lower temperatures (even by a temperature jump on the surface of adipocytes of approximately 10 K), but in this case the sWAT must have very high Young’s modulus. This can be achieved e.g. by mechanical deformation of sWAT with high deformation rates (see Equation (A.9) and the following discussion).

## Conclusion

Heating of sWAT through the application of RF current and consequently the relevant aesthetic effects of such treatment must be strongly dependent on the morphological type of sWAT. Application of RF current to hypertrophic sWAT can produce very high microscopic temperature gradients caused by large differences in electrical conductivities between the peri-cellular sheath of ECM around hypertrophic adipocyte and its volume. Such “channeling” of the current can lead to much higher local temperatures and temperature gradients near adipocytes’ surfaces than was previously assumed.

High temperature gradients can produce thermo-mechanical stresses on the cells’ surfaces. Whereas under normal conditions these stresses are small and cannot cause the fracturing of the cell structure, application of supportive mechanical forces (e.g. of high deformation rates), which can significantly modulate the Young’s modulus of sWAT, increases these stresses by several orders of magnitude, thus increasing the probability of thermo-mechanical cell damage. This effect is much less probable in sWAT of a normal or of a hyperplastic type, where the peri-cellular structures are not developed sufficiently. These differences in a thermo-mechanical behavior of sWAT of different morphological types can be the main reason for high inter-patient dispersion of treatment results after application of RF currents as often observed in clinical applications.

Contrarily, the role of the so-called effect of “collagen shrinkage”, which is traditionally considered to be one of the main effects of RF application not only to the dermis but also to sWAT, is generally over-estimated. The realization of this effect in non-invasive body contouring is very doubtful.

This new formalization of the effect of RF current onto sWAT not only demands the development of some reliable procedure of differential diagnostics between different types of sWAT but also means that the whole treatment strategy of RF application must be thoughtfully revisited.

## Appendix

### Physical Model of sWAT Heating with RF Current

Let us consider a spherical adipocyte of the radius, *R*, surrounded by a peri-cellular fibrotic structure with the thickness, *h*, which in general can be dependent on *R*. The initial temperature inside of adipocyte is *T*_*i*_. External heating with RF current will quickly increase the surface temperature of adipocyte to the value of *T*_*e*_. The corresponding temperature gradient between the cell surface and its volume will produce the heat flow into the cell, causing production of an inhomogeneous temperature field which, because of the cell’s symmetry, will be dependent only on the distance, *r*, from the center of the sphere and on the time of heating, *t*. Such an inhomogeneous temperature field in an adipocyte will cause production of radial and tangential stresses on its surface and/or in its volume. If these stresses reach the critical value of that of tensile strength, σ_*f*_, the cell can rupture. The type of rupturing must depend on the heating conditions and can occur either superficially or deep in the cell’s volume.

The heat equation for a sphere of radius *R* in the case of symmetrical heating can be written in the form:

$$\frac{\partial T\left(r,t\right)}{\partial t}=\varkappa \frac{1}{r^{2}}\frac{\partial }{\partial r}\left(r^{2}\frac{\partial T\left(r,t\right)}{\partial r}\right)-\frac{T\left(r,t\right)}{\tau }+\frac{Q_{v}\left(r,t\right)}{c_{v}},$$ (A.1)

where ϰ is the coefficient of thermal diffusivity, *τ* is the time constant of tissue perfusion, Q_*v*_ is the rate of heat production and *c*_*v*_ is the heat capacity per unit volume. Perfusion can be neglected by short heating times (in this case, *τ* = ∞). We will also neglect the direct heat production inside the cell during RF application, assuming the heating takes place only on the surface of adipocytes, i.e. *Q*_*v*_ (*r*,*t*) = *Q*_*v*_ *δ*(*r—R*). Moreover, we will further consider the case of adiabatic (without heat and mass exchange) heating, which gives us the possibility to neglect the first term in the right-hand side of Equation (A.1). Equation (A.1) has the initial condition *T*(*r*,*0*) = *T*_*i*_ and it can have different boundary conditions corresponding to the art of heating and heat exchange on the surface of the sphere. To make the estimation of temperature gradients, we will now assume the initial heating takes place only in the peri-cellular structure on the cell’s surface during a very short time, Δ*t*.

Adiabatic increase of the temperature on the cell surface, Δ*T*_*R*_, can be assessed from Equation (A.1) as:

$$\Delta T_{R}=\frac{Q_{v}}{c_{v}}\Delta t$$ (A.2)

The rate of the local heat released per unit volume of the tissue during the application of RF current, *Q*_*v*_, can be described by the Joule’s law:

$$Q_{v}=\frac{j^{2}}{\lambda },$$ (A.3)

where *j* is the current density and *λ* is the electrical conductivity of the tissue. Higher current densities and lower electrical conductivities must consequently cause higher release of the heat in the tissue. From here:

$$\Delta T_{R}=\frac{j^{2}}{c_{v}\lambda }\Delta t$$ (A.4)

We will first consider sWAT to be an electrically homogeneous tissue. Whereas the RF current density can significantly vary microscopically, we will not currently take this variation into account and only consider the average current density in the treated area. To make the estimations, we will set this density to be *j* = 0.3 A/cm^2^ = 0.3x10^4^ A/m^2^. The given value of RF density in sWAT is realistic, since the current densities applied to the skin surface in bi-polar RF procedures are usually in the order of 1.0–2.0 A/cm^2^. The average electrical conductivity of sWAT will be set to *λ* = 0.02 S/m [16]. The heat capacity of the fat tissue, *c*_*v*_, can vary between 2.4 J/cm^3^/K [20] and 3.5 J/cm^3^/K; this parameter is known to be isotropic and only slightly temperature dependent [21].

Whereas the absolute average temperature in the tissue increases with the time of RF application, the difference between the temperatures on the surfaces and inside the adipocytes will be limited by the processes of thermal diffusion. Coefficients of thermal diffusion in dermis (similar to a peri-cellular structure around adipocytes) and in a fat tissue are isotropic, independent of the temperature and both have the similar values of ϰ ~ 0.0011 cm^2^/s. Thus we can assume the heat diffusion in such a system will be isotropic and will not predominantly take place along the peri-cellular structures. Taken the critical diffusion length (*l*_*cr*_) in sWAT to be 10 μm, the time of adiabatic heating can be estimated as $\Delta t\sim l_{cr}^{2}/\varkappa \sim$ 0.9 ms. For heating during the time of Δ*t* = 10 ms, the heat diffusion length will be 33 μm, which is the typical adipocyte’s radius. In other words, the RF current during the first several milliseconds of its application will produce the inhomogeneous temperature distribution in sWAT with its highest temperatures occurring at the adipocytes’ surfaces. A longer application of RF current will further increase the average temperature in the tissue; however, the temperature differences outside and inside of adipocytes will not be significantly increased any more.

To make an estimation of the maximum temperature difference between the cell surface and its volume, we will now set the adiabatic heating time to be Δ*t* = 5 ms. Under these conditions and for the current density of *j* = 0.3 A/cm^2^, the homogeneous temperature increase in sWAT can be estimated from Equation (A.4) to be Δ*T*_*R*_ ~ 0.7 K. This value will be only 2.0 K, even for the higher current density of *j* = 0.5 A/cm^2^, which is not sufficient to produce any structural modifications of sWAT. These estimations were made under assumption of the homogeneous distribution of the current density in the tissue. However, if the current flow is mainly limited to a peri-cellular structure building the thin layer, *h*, around the adipocyte of radius, *R*, it is easy to show that for *h << R* the cross-section of the current flow will be reduced by the factor of 2*h / R*. In this case the effective cross-section of *π*(*R* + *h*)^2^ for homogeneous current flow through one adipocyte with a peri-cellular structure around it must be replaced by the cross section of *π*[(*R* + *h*)^2^—*R* ^*2*^] corresponding to the inhomogeneous pathway of RF current, exclusively through the peri-cellular structure of this adipocyte. Therefore, the current density near the adipocyte’s surface will be increased by the factor *R/*2*h*, producing the effect of RF “channeling”. For *R* = 40 μm and *h* = 2 μm, this means the increase of the current density near the cell surface by 10 times compared to the average current density used in our previous estimations. At the same time, according to Equation (1) the electrical conductivity in Equation (A.4) must be in this case changed from *λ* to *λ*_*s*_ ~ 0.34 S/m. Furthermore, we will neglect the difference in heat capacitances between the peri-cellular structures and the triglycerides. According to (A.4), the RF channeling can cause an almost 6 times higher temperature increase in a peri-cellular area, which can now reach the value of about 4 K and 12 K by current densities in sWAT of *j* = 0.3 A/cm^2^ and of *j* = 0.5 A/cm^2^, respectively. One can conclude that the effect of current channeling in the peri-cellular area of adipocytes will lead to a temperature increase which can indeed significantly influence the structure and stability of adipocytes.

These estimations are critically dependent on the coefficient of channeling, *R/*2*h*. The ratio between the adipocytes’ radius and the thickness of its peri-cellular structure is not known. It is however well-recognized that these structures are much more developed in hypertrophic sWAT containing larger adipocytes than in other types of sWAT. If the thickness of the peri-cellular structure increases linearly with *R*, the coefficient of current channeling, *R /* 2*h*, will be the same for all adipocytes. If, however, the thickness of the peri-cellular structure increases slower than the cell radius, *R*, which can be assumed to be the case in a real sWAT, this coefficient will be higher for the large adipocytes. This phenomenon can make the large adipocytes preponderant for the electrical conductivity in sWAT, producing additional inhomogeneity of the temperature field in sWAT under RF application.

### Thermo-Mechanical Model of RF Application

By continuous application of RF current of the same current density for longer than the adiabatic time, the average temperature in sWAT will further increase, but the temperature difference between peri- and intra-cellular areas of adipocyte will be approximately of the same value as it was during the adiabatic phase of heating. Rapid increase of the temperature in a peri-cellular area of adipocytes during the adiabatic phase of RF application will not only cause the production of high microscopic temperature gradients near adipocytes’ surfaces, but can also produce a significant thermo-mechanical stress in this area. Radial, σ_*r*_, and tangential, σ_θ_, stresses of thermo-elastic nature in a spherical cell are connected through an equation of mechanical equilibrium:

$$\frac{d\sigma_{r}}{dr}+\frac{2}{r}\left(\sigma_{r}-\sigma_{\theta }\right)=0$$ (A.5)

The corresponding solution for radial and tangential stresses caused by inhomogeneous temperature distribution in a sphere of the radius *R* is well known and has the form:

$$\sigma_{r}\left(t\right)=\frac{2\alpha E}{1-\nu }\left\{\frac{1}{R^{3}}\overset{R}{\underset{0}{\int }}T\left(r,t\right)r^{2}dr-\frac{1}{r^{3}}\overset{r}{\underset{0}{\int }}T\left(r,t\right)r^{2}dr\right\}$$ (A.6)

$$\sigma_{\theta }\left(t\right)=\frac{\alpha E}{1-\nu }\left\{\frac{2}{R^{3}}\overset{R}{\underset{0}{\int }}T\left(r,t\right)r^{2}dr+\frac{1}{r^{3}}\overset{r}{\underset{0}{\int }}T\left(r,t\right)r^{2}dr-T\left(r,t\right)\right\}$$ (A.7)

Here, *α* is the coefficient of thermal expansion, *E* is the Young’s modulus of elasticity and *v* is the Poisson’s ratio. If *T*(*r*,*t*) is known from Equation (A.1), one can calculate from Equations (A.6) and (A.7) the mechanical stresses produced in the cell.

Let us consider the case of a cell with an initial internal temperature of *T*_*i*_ which surface is quickly heated to the temperature, *T*_*e*_. This could realistically be the case for sWAT heating through the application of RF current during a short time of several milliseconds. The maximum compressive stresses occur in this case on the cell’s surface at very early times of heating and they have the value of:

$$\sigma^{max}\left(R\right)=-\frac{\alpha E\left(T_{e}-T_{i}\right)}{1-\nu }$$ (A.8)

A cell will fracture on its surface if *σ* ^*max*^ = *σ*_*f*_. Assuming the linear heat transfer, the maximum temperature jump at the cell surface which will not cause mechanical fracturing of the cell can then be described by the following equation:

$$\Delta T_{max}=\frac{\sigma_{f}\left(1-\nu \right)}{\alpha E}$$ (A.9)

From here, one can assess the temperatures which are needed to produce the thermo-mechanical cell damage.

It is clear that higher values of Poisson ratio (ratio of transverse to axial strain), *v*, will effectively decrease the temperature needed for mechanical damage of the cell. To make the estimations, we will further consider the case of *v* ≅ 1/2, which is typical for the soft tissues [22]. The value of the coefficient of thermal expansion,*α*, is dependent on the material structure and cannot be changed by the orders of magnitude. For example, the value of *α* for triglycerides can vary several times dependent on the weighting of different triglycerides in the mixture as well as on the water content. Furthermore we will set *α* = 8x10^-4^ K^-1^.

To make the final estimation, we must now assess the values of *σ*_*f*_ and *E*. These values can vary in some intervals dependent on the conditions of the material deformation. The value of *σ*_*f*_ was assessed to have the order of magnitude of 10^4^ Pa [4]. A very similar value of *σ*_*f*_ was determined in sWAT of the wild mice with a normal content of Col VI, whereas the same value in Col6KO mice was measured to be only 0.5x10^4^ Pa [6]. For low rates of deformation, the Young’s modulus of sWAT was measured to have the value of 10^3^ Pa [4]. Under these conditions Δ*T*_*max*_ can be estimated from Equation (A.9) to be over 6,000 K. This unrealistically high temperature, which can never be applied clinically, would immediately, deeply burn the tissue. Apparently, this should prove that the mechanism of thermo-mechanical damage cannot be realized in a fat tissue.

The situation can however be very different if the values of the Young’s modulus are much higher than we assumed until now. It is commonly known that the Young’s modulus can reach values of up to 10^6^ Pa by deformation of sWAT with high deformation rates [19]. Under such conditions, the estimation of Δ*T*_*max*_ will give the realistic value of the temperature increase needed for fracturing of approximately 10 K. According to the estimation of the temperature increase in sWAT during the RF application performed above (see Equation (A.4)), this value can be reached by current densities in sWAT of more than 0.5 A/cm^2^. One can conclude that under these conditions a selective temperature increase near the adipocyte’s surface can lead to thermo-mechanical damage of adipocytes.

It must be noted that the change of boundary conditions in Equation (A.1) can modify the value of Δ*T*_*max*_ by some factor but not by the orders of magnitude. At the same time, variation of the heat delivery mechanism (e.g. a constant heat flow through the cell surface instead of a constant cell surface temperature) can shift the maximum stress from the cell’s surface into its volume, thereby initiating another type of the cell damage. These peculiarities will be discussed elsewhere.
